# Let's talk about flux or the importance of (intracellular) reaction rates

**DOI:** 10.1111/1751-7915.12455

**Published:** 2016-11-11

**Authors:** Lars M. Blank

**Affiliations:** ^1^ Institute of Applied Microbiology ‐ iAMBAachen Biology and Biotechnology ‐ ABBtRWTH Aachen UniversityWorringer Weg 152074AachenGermany

The physiology of cells, determined by all extracellular production and consumption rates, intracellular reaction rates and the growth rate, is of fundamental interest in such different fields of the life sciences as microbiology, biotechnology and medical applications. In this Crystal Ball, I emphasize the importance of metabolic reaction rates, that is fluxes, for our understanding of metabolic network operation and envisage a time in which fluxes can be changed at will. Indeed, the ultimate goal of many fields of the life sciences is to understand cell physiology and hence the flux distribution, which characterizes the metabolic state of a cell. Based on this information, it will be possible to design strategies, for example, to avoid certain phenotypes (applications in infection biology and cancer research) or to redirect fluxes (applications in industrial biotechnology to increase rate, yield and titre of a product of interest).

I argue that we are now in the lucky position that we can measure extra‐ and intracellular fluxes, that we have well‐characterized genetic tools for changing enzyme activities, and that we have fermentation capabilities to maintain optimal environmental conditions for a microorganism. Rationally changing a flux, however, requires a solid understanding of how the interplay of all mentioned aspects (and more) result in the observed flux distributions.

Before describing some of these developments, we have to agree on the importance of the goal to precisely manipulate cellular fluxes. In many research projects, fluxes are in fact ignored, despite the ultimate goal implicitly being to understand metabolic network operation. For example, complex interactions between signalling pathways is often studied while ignoring cell physiology, although many cellular processes are growth rate dependent. And in industrial biotechnology, often the titre of the product of interest is communicated as this value quantifies overall performance of the biocatalytic reaction, implicitly even the product production rate (i.e. the flux towards the product) assuming a defined experimental time. Of course, when the fermentation is reported in the context of the entire process, titre is central; as it considers physical parameters including product solubility, the yield of the product on a given substrate and product inhibition of the biocatalyst besides other aspects. However, this is not the only critical value relevant to the process.

In Metabolic Engineering, we propose to execute rational strain engineering, with the performance of the (microbial) cell in focus. Still, often end‐point concentrations (e.g. g L^−1^) and not specific rates normalized to the cell dry weight used (e.g. g_product_ per g_CDW_ per hour) are reported. When manipulating enzyme activities (e.g. using a stronger promoter or a more active enzyme variant), one implicitly aims for a change in flux at a given network node; the subsequent outcome of this flux change being a change in product concentration. A focus on end‐point assays potentially misses interesting clones that show a significant improvement in production rate, hence achieve the maximal titre long before the end‐point. However, there currently is no facile method to observe cellular fluxes on a broad scale.

A potential answer discussed in the literature to the challenge of measuring fluxes *in vivo* is the application of *in vivo* metabolite sensors that respond to metabolite concentrations using fluorescent reporter systems. Cells carrying these sensors can be cultivated under constant fluorescence measurements conditions. A wide variety of fluorescent reporters is available, so metabolite sensors that report intracellular concentrations via fluorescence can be used for an ever increasing number of metabolites. It seems likely that we will see ever more advanced sensors with, for example, increased linear dynamic range for the analyte‐to‐sensor signal, lower interference from host components and lower interference of host metabolism. However, it has to be clear that these sensors measure first of all concentrations and not fluxes! In combination with a dedicated host that has a defined flux for metabolite export, such a novel sensor might even report flux changes non‐invasively in real time.

The ability to sense intracellular fluxes may in fact be a native ability of cells in general. The concept of intrinsic flux sensors as a means to allocate resources in the metabolic network was postulated for *Escherichia coli* (Kochanowski *et al*., 2013). Also in *Saccharomyces cerevisiae* and other yeasts, the rate of glucose uptake strongly influences ethanol production and the flux through the TCA cycle (Blank *et al*., 2005; Heyland *et al*., 2009), suggesting the presence of a flux sensing mechanism.

Determining the flux through the TCA cycle and ideally the entire flux distribution in (central carbon) metabolism may be simple or almost impossible, depending on the cell and environment studied. Pathway redundancy and the lack of experimental measurements, which could be otherwise used to close metabolite mass balances might hamper identifiability of particular fluxes. For example, the flux through glycolysis in lactic acid bacteria (a linear pathway from glucose to lactate) can be resolved by simple flux balancing, while aerobic glucose metabolism in *E. coli* requires advanced ^13^C‐based metabolic flux analysis (Blank and Kuepfer, 2010). In most aerobic bacteria grown on sugars, the flux distribution through a genome scale metabolic network can only be partially resolved; despite this, the available flux analysis tools (e.g. 13C Flux2, Weitzel *et al*., 2013) are an important aid in our Metabolic Engineering efforts. Examples for identifying metabolic limitations by flux analysis are limitations in the redox cofactor regeneration rates of NADH (Blank *et al*., 2008) and NADPH (Becker *et al*., 2007).

With the concepts of Synthetic Biology, orthogonality, modularity, but foremost standardization, we can finally progress towards truly rational strain engineering. The rate of transcription and translation can be changed significantly and – importantly – in a generally predictable manner. For example, the affinity of the ribosome via the 16S rRNA to ribosome binding sites was computed, promoters characterized in detail, and other genetic elements were introduced to deliver standardized tools that facilitate flux rerouting (reviewed, e.g. in Blank and Ebert, 2013). For example, the development of the Standard European Vector Architecture speeds up the genetic tool construction. The shared tools (SEVA, http://seva.cnb.csic.es/) give an indication of the effort of the research community. One has not to be too optimistic to state that we are slowly but surely entering the area of writing the genetic code, as compared to only reading it.

The many aspects that ultimately define the flux distribution in a given metabolic network are so complex that considerable effort is still needed to achieve, for example, a significantly increased glucose uptake rate (actually a good idea in many whole‐cell catalysts, as the substrate uptake rate determines the maximal achievable specific flux towards the product and influences the space time yield, e.g. g_product_ per L_reactor_ per hour). While many strategies including transporter overexpression failed, 15 years ago Peter Ruhdal Jensen and colleagues reported an *E. coli* with a 1.7‐fold increased glucose uptake rate (Koebmann *et al*., 2002). The authors simply created a demand for ATP, to which *E. coli* responded very drastically. Recently, we used the postulated ‘driven‐by‐demand’ concept in my laboratory for the synthesis of rhamnolipids (a biosurfactant with rhamnose as hydrophilic and two beta‐OH‐fatty acids as hydrophobic moieties) in *Pseudomonas putida* (Tiso *et al*., 2016). *P. putida* can sustain very high sugar uptake rates under stress conditions (e.g. in the presence of a second octanol phase), but notably produces only biomass and CO_2_, with no ‘overflow’ metabolites encountered (Blank *et al*., 2008) as in other popular hosts like *E. coli*,* Bacillus subtilis* and *S. cerevisiae*. Creating a demand for activated rhamnose (i.e. dTDP‐rhamnose) and activated beta‐OH‐fatty acids (i.e. ACP‐fatty acid) resulted in a strain with 55% theoretical yield of rhamnolipid on sugar, a flux increase of 300% in the dTDP‐rhamnose pathway, and 50% increase in *de novo* fatty acid synthesis.

Considering fluxes in many more biotechnological projects and even life science research in general will certainly yield great rewards, and many surprises are waiting for us. We have many of the tools at hand, and are developing many more; now, we must use them to their full effect to illuminate metabolism.
