# Let's talk about flux or the importance of (intracellular) reaction rates

**DOI:** 10.1111/1751-7915.12740

**Published:** 2017-06-22

In the original publication (*Microbial Biotechnology*, 2017; 10(1): 28–30), the funding information was omitted. The funding information should read as follow:

‘Research at the laboratory of Lars M. Blank is besides other sources supported by grants from the European Union's Seventh Framework Programme under Grant agreement no. 602156, and the Horizon 2020 research and innovation Programme under Grant agreement no. 633962. Also, research is supported by the German Federal Ministry of Education and Research (BMBF) as part of the ERA‐SynBio project SynPath and the e:Bio project YeastScent. In addition, the Excellence Initiative of the German federal and state governments as a part of the Cluster of Excellence ‘Tailor‐Made Fuels from Biomass’ (TMFB) is acknowledged. The scientific activities of the Bioeconomy Science Center are supported financially by the Ministry of Innovation, Science and Research within the framework of the NRW Strategieprojekt BioSC (No. 313/323‐400‐002 13)’.

We sincerely apologize for the confusion caused by this error.

## References

[mbt212740-bib-0001] Blank, L.M. (2017) Let's talk about flux or the importance of (intracellular) reaction rates. Microb Biotechnol 10: 28–30. 27863005 10.1111/1751-7915.12455 PMC5270755

